# Deep Learning Provides a New Magnetic Resonance Imaging-Based Prognostic Biomarker for Recurrence Prediction in High-Grade Serous Ovarian Cancer

**DOI:** 10.3390/diagnostics13040748

**Published:** 2023-02-16

**Authors:** Lili Liu, Haoming Wan, Li Liu, Jie Wang, Yibo Tang, Shaoguo Cui, Yongmei Li

**Affiliations:** 1 Department of Radiology, The First Affiliated Hospital of Chongqing Medical University, No. 1 Youyi Road, Yuzhong District, Chongqing 400016, China; 2 Department of Radiology, Chongqing General Hospital, Chongqing 401120, China; 3 College of Computer and Information Science, Chongqing Normal University, Chongqing 400016, China; 4 Department of Radiology, The People’s Hospital of Yubei District of Chongqing, Chongqing 401120, China; 5 Department of Nuclear Medicine, The First Affiliated Hospital of Chongqing Medical University, Chongqing 400016, China

**Keywords:** high-grade serous ovarian cancer, deep learning, magnetic resonance imaging

## Abstract

This study aims to use a deep learning method to develop a signature extract from preoperative magnetic resonance imaging (MRI) and to evaluate its ability as a non-invasive recurrence risk prognostic marker in patients with advanced high-grade serous ovarian cancer (HGSOC). Our study comprises a total of 185 patients with pathologically confirmed HGSOC. A total of 185 patients were randomly assigned in a 5:3:2 ratio to a training cohort (n = 92), validation cohort 1 (n = 56), and validation cohort 2 (n = 37). We built a new deep learning network from 3839 preoperative MRI images (T2-weighted images and diffusion-weighted images) to extract HGSOC prognostic indicators. Following that, a fusion model including clinical and deep learning features is developed to predict patients’ individual recurrence risk and 3-year recurrence likelihood. In the two validation cohorts, the consistency index of the fusion model was higher than both the deep learning model and the clinical feature model (0.752, 0.813 vs. 0.625, 0.600 vs. 0.505, 0.501). Among the three models, the fusion model had a higher AUC than either the deep learning model or the clinical model in validation cohorts 1 or 2 (AUC = was 0.986, 0.961 vs. 0.706, 0.676/0.506, 0.506). Using the DeLong method, the difference between them was statistically significant (*p* < 0.05). The Kaplan–Meier analysis distinguished two patient groups with high and low recurrence risk (*p* = 0.0008 and 0.0035, respectively). Deep learning may be a low-cost, non-invasive method for predicting risk for advanced HGSOC recurrence. Deep learning based on multi-sequence MRI serves as a prognostic biomarker for advanced HGSOC, which provides a preoperative model for predicting recurrence in HGSOC. Additionally, using the fusion model as a new prognostic analysis means that can use MRI data can be used without the need to follow-up the prognostic biomarker.

## 1. Introduction

The majority of deaths from gynecological cancer are caused by ovarian cancer. High-grade serous ovarian cancer (HGSOC) is the most prevalent and lethal histological form, accounting for 70% of all fatalities [1,2,3]. At the time of therapy, 60% of ovarian cancer patients were in an advanced state [3]. Currently, procaine-platinum chemotherapy, followed by tumor cell reduction, is the principal course of treatment for patients with HGSOC [1,4]. This course of treatment is beneficial for roughly 80% of HGSOC patients [5]. The median progression-free survival (PFS) of patients with advanced HGSOC patients is just 18 months, and most HGSOC patients still experience tumor recurrence. Recurrence is thought to be the primary factor in these individuals’ deaths [6,7,8]. Therefore, it is crucial to anticipate the recurrence of patients with advanced HGSOC in order to develop a precise and individualized treatment strategy and extend the patients’ survival time. Predicting postoperative recurrence is currently difficult. To enhance the prognosis of these individuals, prognostic indicators for advanced HGSOC must be developed. The diagnosis of ovarian disorders frequently uses magnetic resonance imaging (MRI). Some of its benefits include high soft-tissue resolution, multi-planar and multi-parameter imaging, non-invasiveness, radiation-free, and reasonable pricing. It also gives additional insight into the prognosis of HGSOC [9,10]. Deep learning, an artificial intelligence technique, has produced positive outcomes in the detection of important features from medical images [11,12,13,14]. Numerous studies in the field of medicine have used deep learning with considerable results [15,16,17,18,19,20]. Through convolutional operations and a hierarchical neural network topology, deep learning extracts tumors’ intrinsic properties, which has strong prognostic value [21].

In this retrospective study, we investigated the deep learning technique to extract the prognostic biomarkers of HGSOC from preoperative MRI scans in order to provide a non-invasive individualized HGSOC recurrence prediction model. Additionally, a novel prognostic analysis approach is provided by the deep learning method, which can only identify prognostic indicators from tumor image data. This allows us to use a vast amount of data without further analysis. Overall, these data may offer crucial insights into designing personalized treatments and post-treatment planning.

## 2. Materials and Methods

### 2.1. Patients

The 185 patients with surgically and pathologically confirmed HGSOC who underwent pelvic MRI exams at the First Affiliated Hospital of Chongqing Medical University between January 2013 and December 2019 were retrospectively examined.

The inclusion criteria were: (1) Stages III or IV of HGSOC, as determined by pathology, according to the International Federation of Obstetrics and Gynecology; (2) the initial tumor cell reduction operation being performed on all patients; (3) pelvic MRI being carried out prior to surgery; (4) follow-up information being available (including MRI and tumor markers); (5) platinum with taxol medicines being used in postoperative chemotherapy.

The exclusion criteria were: (1) preoperative increasing cancer antigen 125(CA-125), human epididymis protein 4(He4), age, or FIGO staging being absent; (2) recurrence within 2 months of the decrease in the initial tumor cells; (3) there being no follow-up for less than 18 months; (4) patients having treatment prior to their baseline MRI; (5) clear MRI picture artifacts; and (6) patients with other cancers besides HGSOC, such as breast cancer.

To achieve the best possible cytoreduction in all pelvic, abdominal, and retroperitoneal lesions, all included patients with advanced HGSOC (n = 185) received primary debulking surgery performed by skilled gynecologic oncologists.

Every patient was checked on every two to four months for the first two years, then every three to six months starting in year three, and then annually starting in year five [22].

Recurrence was the study’s endpoint, and it was determined by combining radiological findings, CA-125 levels, and clinical symptoms. Recurrence-free survival (RFS) is the period of time between clinical remission and the first recurrence [23]. Age, preoperative CA-125, HE4, residual tumor status, FIGO stage, tumor location and maximum tumor diameter, metastases, Ki67, and progesterone receptors (PR) were among the preoperative clinical parameters that were gathered from the institutional picture archiving and communication system (PACS) (Table 1).

### 2.2. MRI Parameters

Two separate scanners (GE, Signa HDxt 1.5 T, and Siemens, MAGNETOM Skyra 3.0 T) were used for the MRI exams. Prior to the examination, the patient had to fast for at least 4 h and fill their bladder with moderate amounts of water. The 1.5 T scanning parameters were as follows: T2FSE:TR/TE 6680 ms/130 ms; slice thickness, 4 mm; gap, 1 mm; field of view, 35–40 cm; DWI (TR/TE, 7000 ms/77.5 ms), b value, 1000 s/mm^2^; and contrast-enhanced T1WI (LAVA, T1CE):TR/TE, 4.2 ms/2.1 ms) gadopentetate dimeglumine (Magnevist, Bayer Schering) was injected at a rate of 2.0 mL/s for the contrast-enhanced pictures. The 3.0 T scanning parameters were as follows: T2FSE:TR/TE 3000 ms/87 ms; slice thickness, 5 mm; field of view, 35 cm; DWI (TR/TE, 5700 ms/92 ms), b value, 800 s/mm^2^; and contrast-enhanced T1WI (VIBE, T1CE):TR/TE 3.5 ms/1.4 ms; slice thickness, 2 mm; gadopentetate dimeglumine (Magnevist, Bayer Schering) was injected at a rate of 2.0 mL/s and then repeated at 25–30, 60–90, and 180 s into the examination.

### 2.3. Development of the Deep Learning Network

We proposed a novel neural network based on ResNet34-CBAM to extract internal features from preoperative MRI images of ovarian cancer patients and assess them from several perspectives in conjunction with clinical data. Preoperative MRI imaging and relevant clinical features of patients can be used to predict the suggested model.

**Figure 1:**
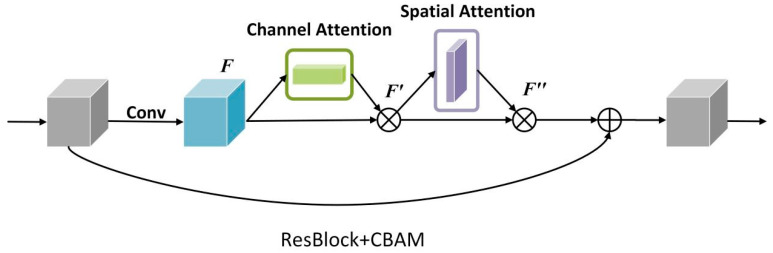
The structure of ResNetCBAM.

**Figure 2:**
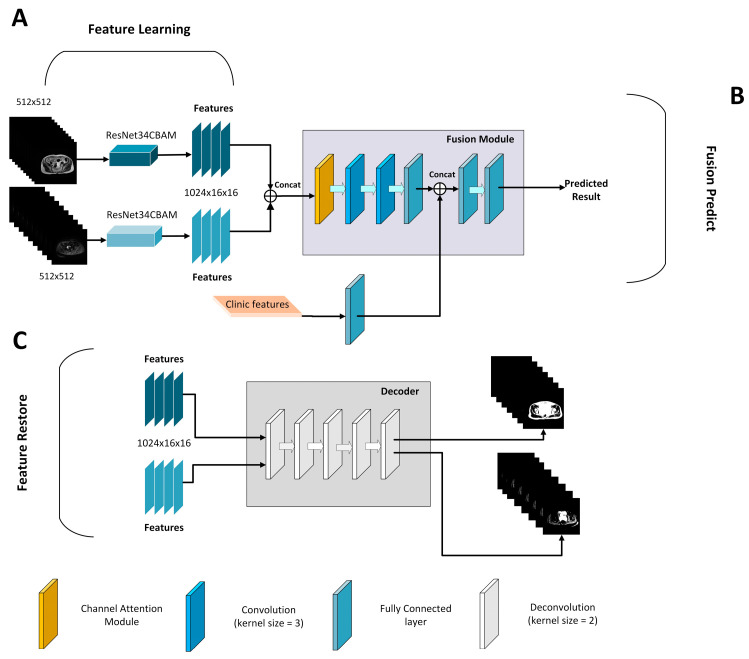
The framework of the fusion model is divided into three parts: (**A**) feature learning, (**B**) feature restoration, and (**C**) fusion prediction. The feature learning part is ResNet34-CBAM structure, which automatically learns MRI image features. The feature restoration part is a decoder structure, mainly for up sampling operation, which is used to restore the acquired image features to images. The fusion prediction part is to fuse the acquired image features with clinical features for prediction.

Dr. He Kaiming suggested ResNet [24] in 2016. The model employs residual connections to address the degradation phenomena that occur as the depth of the deep neural network increases; as the network depth increases, the network’s accuracy saturates or even falls. The residual connection supplements the possible loss in image feature extraction and eliminates the problem of the depth neural network is too deep to learn by adding the extracted image features to the original input. ResNet CBAM extends ResNet’s capacity to learn images by incorporating channel attention and spatial attention (Figure 1). The original ResNet, as the entire connection layer of the classifier, including the convolution layer used to extract image features, removes redundant features, compresses the pooling layer of data, and finally combines features and maps the feature space to the tag space. Next, it accelerates convergence speed, reduces internal variable transfer, add a batch normalization layer between layers, and use the adaptive average pooling layer to compress spatial dimensions before finally entering the full connection layer, while filtering redundant features and reducing the number of model parameters. Based on this premise, ResNet CBAM includes channel attention and spatial attention to learn the significant aspects of the characteristics collected by ResNet. As a result, we used ResNet34-CBAM as the feature extraction to learn the image features automatically. Simultaneously, to verify whether the extracted features showed the inherent features of the image tumor, we used the decoder’s deconvolution layer to reconstruct the extracted features into an image, which we compared and evaluated with the original image. To achieve this goal, we trained the ResNet34CBAM using 185 patients. Radiologists evaluated tumors on all MRI slices from 185 patients, resulting in 3839 tumor pictures to train the network. During the training process, the network was iteratively optimized until the intrinsic image features were extracted. After obtaining the features of two modes via ResNet34-CBAM, the clinical features were entered into the information fusion device to fuse clinical features and predict HGSOC (Figure 2). The information fusion device has two convolution layers, two linear activation layers, one channel attention layer, and one full connection layer for final classification.

**Figure 3:**
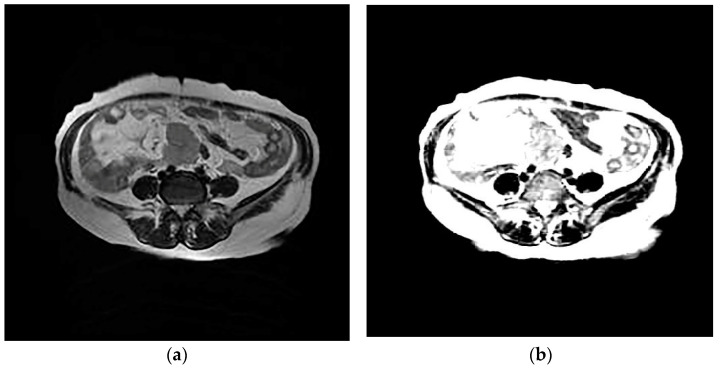
Axial T2-weighted image (**a**); generated image (**b**).

**Figure 4:**
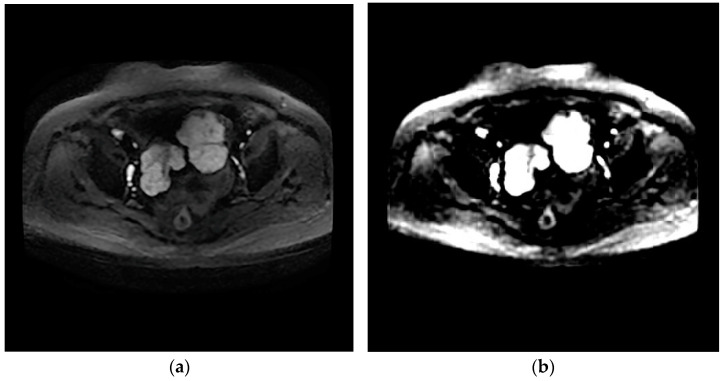
Axial DWI image (**a**); generated image (**b**).

The design of the network can provide the value of image features. The network eliminates redundant information through learners and decoders, and only retains features that can reflect the inherent characteristics of HGSOC, and therefore a small number of features can contain most of the information of tumor images. Therefore, we input T2WI and DWI images from the same patient into the network and reconstructed them into images. It was found that the reconstructed images based on the features were similar to the original images, which proves that the internal features of the images are learned, as shown in Figure 3 and Figure 4.

### 2.4. Deep Learning Feature Extraction

When the trained neural network was obtained, it was used to extract the internal features of the image from MRI images of patients with high-grade serous ovarian cancer. First, we chose T2WI and DWI sequences from MRI images of patients with tumor areas, entered them into the network according to patient number, and used bilinear interpolation to resize them all to 512 × 512 before input. We then calculated the mean and variance of these images and normalized the mean and variance. They were then routed to the RestNet34CBAM network for feature extraction. We averaged the features of the same patient’s image slices in the same sequence to obtain the T2WI modal image features and DWI modal image features corresponding to each patient, and then sent the two features to the information fusion device because each patient’s sequence contained multiple tumor slices.

### 2.5. Clinical Characteristics

Because clinical features were employed as prognostic biomarkers in HGSOC [25], we compared the prognostic significance of the deep learning feature with the clinical characteristics. We developed a clinical model using features such as age, preoperative CA-125, HE4, residual tumor state (RD), FIGO stage, tumor location, maximum tumor diameter, ascites, lymph node metastasis, peritoneal metastasis, distant metastasis, KI-67, and PR. We converted these clinical features into numerical values and then combined them into a vector with the patient number to facilitate one-to-one correspondence with the patient image features. We standardized the clinical features of all patients with a z-score after obtaining the clinical feature vector, and then fused them with the extracted image features. At the same time, we used a deep confidence network to screen the clinical features. A clinical model was built based on the clinical characteristics to predict the recurrence probability.

## 3. Statistical Analyses

Age, maximum tumor diameter, preoperative HE4 levels, and CA-125 levels were all reported, along with their medians and interquartile ranges (IQRs). Other categorical variables were presented in the form of frequencies and proportions. The F-test/independent samples *t*-test and Fisher’s exact test, respectively, were used to examine differences in continuous variables and categorical variables. Every statistical test had two sides. The cutoff for significance was *p* < 0.05.

A threshold of 0.5 was used to categorize the prediction scores produced by each model in order to assess the reliability of the clinical model, the deep learning model based on the fusion of T2WI and DWI images, and the fusion model based on clinical features and deep learning features. The risk of recurrence was high for values above 0.5 and low for values below 0.5. At the same time, we assessed the consistency between the model’s predicted recurrence probability and the actual recurrence using Harrell’s consistency index. The Kaplan–Meier chart was used to assess the correlation between the risk score of the prediction model and patients’ RFS. The index was between 0.6 and 0.8, indicating that the model was effective. To assess the model’s performance and accuracy, we used the AUC value of the area under the receiver operating characteristic curve (ROC curve) and the corresponding 95% CI, as well as the accurate value and the corresponding 95% CI. The DeLong method was used to test the statistical differences between ROC curves [26]. The model proposed in this study was built with Python programming software (version 3.9 Python) and the lifelines package in Python.

## 4. Results

### 4.1. Patient Characteristics

The basic characteristics of all patients in our dataset are summarized in Table 1. Preoperative CA125, HE4, maximum tumor diameter, tumor location, residual status of tumor, peritoneal metastasis, and PR were the clinical characteristics with statistical differences between the recurrence and non-recurrence groups.

### 4.2. Deep Learning

In the two validation sets, the fusion model had the best prediction effect (validation set 1 C-index = 0.752, [95% CI: 0.734–0.770], validation set 2 C-index = 0.813, [95% CI: 0.782–0.844]), followed by the deep learning model (validation set 1 C-index = 0.625, [95% CI: 0.582–0.668], validation set 2 C-index = 0.600, [95% CI: 0.554–0.644]), The clinical characteristic model showed the worst performance (validation set 1 C-index = 0.505, [95% CI: 0.457–0.552], validation set 2 C-index = 0.501 [95% CI: 0.447–0.555]) (Table 2).

**Figure 5:**
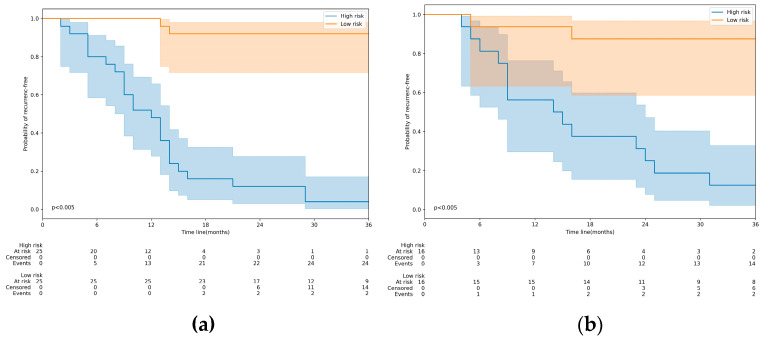
Kaplan–Meier’s analysis of the Fusion model. Kaplan–Meier’s analysis in patients from the validation set 1 (**a**). The vertical lines indicate censored data, and the shadow indicates the 95% confidence interval. Kaplan–Meier’s analysis in patients from the validation set 2 (**b**).

Furthermore, the Kaplan–Meier analysis confirmed the strong correlation between the model’s prediction score and RFS (Figure 5). With a prediction score of 0.5 as the threshold, we divided the patients into high- and low-risk recurrence groups and found significant differences in RFS between the two groups in both validation sets.

At the same time, we calculated the AUC value of the model on the two validation sets to further demonstrate the fusion model’s predictive ability for recurrence, and the fusion model reached 0.986 (95% CI: [0.977–0.995]) on validation set 1 and 0.961 (95% CI: [0.937–0.985]) on validation set 2, proving that the fusion model had a good predictive ability for recurrence.

In the two validation sets, the AUC of the deep learning model was higher than that of the clinical model (validation set 1 AUC = 0.706, [95% CI: 0.655–0.757]; validation set 2 AUC = 0.676, [95% CI: 0.630–0.722]); however, the fusion model’s AUC was significantly higher than those of the clinical model and the deep learning model.

Using the DeLong method, the difference between the models was statistically significant (*p* < 0.05). (Clinical model vs. deep learning model, *p* = 0.00349; clinical model vs. fusion model, *p* = 0.00001; deep learning model vs. fusion model, *p* = 0.00127).

## 5. Discussion

To our knowledge, only a few studies on MRI-based deep learning for recurrence prediction in high-grade serous ovarian cancer have been published. As a result, the initial goal of our study was to create and evaluate a fusion model based on MRI features and clinical data to predict the probability of recurrence in HGSOC patients. Our findings suggest that the fusion model may be more useful than the clinical model in predicting RFS in patients with advanced HGSOC. Furthermore, the fusion model had a higher AUC than models based on images alone or clinical characteristics alone.

Some clinical trials have demonstrated that procaine–platinum treatment can extend the RFS of patients with advanced HGSOC and increase patient survival [27,28,29]. The clinical needs of patients who are at high risk of recurrence for such treatment have not been satisfied. In clinical practice, however, no possible indicators have been shown to predict HGSOC recurrence. As a result, effective tumor recurrence biomarkers will enable personalized treatment of advanced HGSOC.

Thousands of neuron routes are used in the deep learning network to extract the inherent properties of HGSOC. ResNet, a classic deep learning network, has long been demonstrated to have a strong ability to self-learn features on a variety of image datasets. In recent years, attention has emerged as an effective learning method. It can make neural networks pay more attention to points of interest while learning features, thereby improving neural network feature learning ability. ResNet CBAM is formed from the combination of the two, which also has a stronger learning ability. The network we created employs ResNet34-CBAM as a learner to extract multi-level features of tumor images from MRI images of HGSOC patients. Different image features are extracted from each layer of the network, beginning with low-level features and progressing to complex high-level features as the level deepens.

The current research demonstrated that, compared to the AUCs from each MRI sequence separately, the combined model from two sequences had a significantly higher AUC for predicting the risk of recurrence. This result is consistent with earlier research that found multi-parametric models to have better predictive performance in a number of carcinomas, such as nasopharyngeal, breast, pancreatic, and rectal carcinomas [30,31,32,33]. Comparatively to the modality of CT, multi-parametric MRI has obvious advantages for offering potential predictors [4]. More significantly, some advanced HGSOCs frequently manifest as small lesions with hazy borders and have a propensity to spread through widespread metastases, making it challenging to distinguish them from primary ovarian masses [34,35]. The performance of the fusion model combining deep learning features and clinical information was significantly higher than that of a single clinical model. This result is consistent with the research of Wang et al. [36].

Although the results are encouraging, our research still has some limitations. First, the ResNe34-CBAM network was used to extract the internal features of MRI images and restore them to images. However, the restored images were similar to the original images on the whole, but there was still some blurring in the fine parts. Later, more detailed restored images can be optimized. At the same time, the specific relationship between the extracted features and the actual development of cancer has not been discussed. Secondly, the performance gap between the single clinical model and the single image model is larger than that of the fusion model, and it is not possible to identify whether clinical features or image features contribute greatly to the combined model. In the future, we can fuse clinical features and image features with different weights in this regard, and then evaluate the important party. Thirdly, due to the limitation of data, only the probability of recurrence of HGSOC can be predicted, but the recurrence time and degree cannot be estimated roughly. In the future, the recurrence of HGSOC can be predicted more carefully and deeply by expanding the data.

## 6. Conclusions

The current study demonstrates that deep learning can provide new MRI-based prognostic biomarkers related to HGSOC recurrence that are more predictive than clinical features. We also created a noninvasive fusion model to predict HGSOC recurrence using preoperative MRI imaging, with the goal of assisting with individualized HGSOC treatment and monitoring plans. In addition, we propose a new method for mining HGSOC’s intrinsic features. This method can use a large amount of data without requiring additional information.

## Figures and Tables

**Table 1:** Characteristics of the 185 Patients in Our Dataset.

Characteristic	Recurrence	No Recurrence	*p*-Value
Age	53.54 ± 9.64	53.13 ± 8.67	0.619
CA125	2975.53 ± 3137.10	820.86 ± 919.41	<0.001
HE4	1134.10 ± 1377.33	301.71 ± 423.21	<0.001
Maximum tumor diameter	8.96 ± 3.49	7.31 ± 3.01	0.001
Location			<0.001
Unilateral	27	42	
Bilateral	82	34	
Residual tumor status			<0.001
R0	0	27	
No R0	109	48	
FIGO stage			0.245
III	99	73	
IV	10	3	
Peritoneal metastasis	95	4	<0.01
Distant metastasis	9	3	0.365
Lymph node metastasis	83	67	0.056
ascites	103	50	<0.001
Ki67			0.55
PR	48	51	0.003

Note: CA125, HE4, Maximum tumor diameter, Location, Residual tumor status, Peritoneal metastasis, ascites, and PR were statistically different between groups with Recurrence and No Recurrence (all *p* < 0.05).

**Table 2:** Prediction Effectiveness of Three Models in Two Validation Sets.

Models	Dataset	C-Index (95% CI)	ACC (95% CI)	AUC (95% CI)
**Clinic Model**	validation set 1	0.505 (0.457, 0.552)	0.539 (0.308, 0.769)	0.506 (0.437, 0.575)
	validation set 2	0.501 (0.447, 0.555)	0.489 (0.250, 0.727)	0.506 (0.430, 0.582)
**Image Model**	validation set 1	0.625 (0.582, 0.668)	0.670 (0.620, 0.720)	0.706 (0.655, 0.757)
	validation set 2	0.600 (0.554, 0.644)	0.651 (0.606, 0.697)	0.676 (0.630, 0.722)
**Fusion Model**	validation set 1	0.752 (0.734, 0.770)	0.940 (0.920, 0.960)	0.986 (0.977, 0.995)
	validation set 2	0.813 (0.782, 0.844)	0.849 (0.788, 0.910)	0.961 (0.937, 0.985)

**Note:** Among the three models in the two validation sets, the fusion model has the best prediction effect.

## Data Availability

The data presented in this study are available on request from the corresponding author. The data are not publicly available due to privacy or ethical considerations.

## Abbreviations

HGSOC	high grade serous ovarian cancer
MRI	magnetic resonance imaging
PFS	progression-free survival
T2WI	T2-weighted images
DWI	Diffusion weighted images
RD	residual tumor state; progesterone receptor
PACS	Picture Archiving and Communication System
CA-125	cancer antigen 125
HE4	Human epididymis protein 4
